# Nanotechnology and multipotent adult progenitor cells in Reparative Medicine: therapeutic perspectives

**DOI:** 10.31744/einstein_journal/2018RB4587

**Published:** 2018-11-29

**Authors:** Angela Mazzeo, Enrico Jardim Clemente Santos

**Affiliations:** 1 Instituto Israelita de Ensino e Pesquisa Albert Einstein, Hospital Israelita Albert Einstein, São Paulo, SP Brazil; 2 Celltrovet, São Paulo, SP Brazil

**Keywords:** Nanotechnology, Stem cells, Therapeutics, Nanotecnologia, Células-tronco, Terapêutica

## Abstract

The biology of stem cells is one of the most dynamic and promising fields of the biological sciences, since it is the basis for the development of organisms. Its biological complexity demands efforts from several lines of research aimed mainly at its therapeutic use. Nanotechnology has been emerging as a new field of study, which shows great potential in the treatment of various diseases. This new area of health has been called “Nanomedicine” or “Bionanotechnology”, which can be applied in Medicine by transport and drug delivery systems, robotic tools to be used in diagnostic and surgical processes, nanobiomaterials, gene therapies, nanobiomedical devices, among others. Because stem cells and Nanotechnology are two areas of extremely promising science, a new field of study, called “stem cell Nanotechnology”, has gradually emerged. In this, Nanotechnology is used to help the stem cells apply their therapeutic potential in the treatment, cure, and repair of the damaged tissues, in an effective and safe way. In this way, stem cell Nanotechnology has generated great interest, since it may result in significant contributions to Regenerative Medicine and tissue engineering. The present work aims to present the state-of-the-art regarding its therapeutic use in Human Medicine.

## RESUMO

A biologia das células-tronco é um dos campos mais dinâmicos e promissores das ciências biológicas, pois é a base do desenvolvimento dos organismos. Sua complexidade biológica demanda esforços de diversas linhas de pesquisa, visando principalmente à sua utilização terapêutica. A Nanotecnologia surge como um novo campo de estudo, o qual demonstra grande potencial no que tange ao tratamento de diversas doenças. Esta nova área da saúde vem sendo denominada “Nanomedicina” ou “Bionanotecnologia”, a qual pode ser aplicada na Medicina por meio da utilização de sistemas de transporte e liberação de fármacos, ferramentas robóticas a serem utilizadas em processos de diagnóstico e cirurgia, nanobiomateriais, terapias gênicas, dispositivos nanobiomédicos, entre outros. Em razão das células-tronco e a Nanotecnologia serem duas áreas da ciência extremante promissoras, um novo campo de estudo, denominado “Nanotecnologia das células-tronco”, surge gradativamente. Neste, a Nanotecnologia é utilizada de forma a auxiliar as células-tronco a exercerem seu potencial terapêutico no tratamento, na cura e na reparação dos tecidos lesionados, de forma eficaz e segura. A Nanotecnologia das células-tronco tem gerado grande interesse, podendo resultar em contribuições significativas na Medicina Regenerativa e na engenharia de tecidos. O presente trabalho teve por objetivo apresentar o estado da arte visando à sua utilização terapêutica na Medicina Humana.

## INTRODUCTION

**Figure 1:**
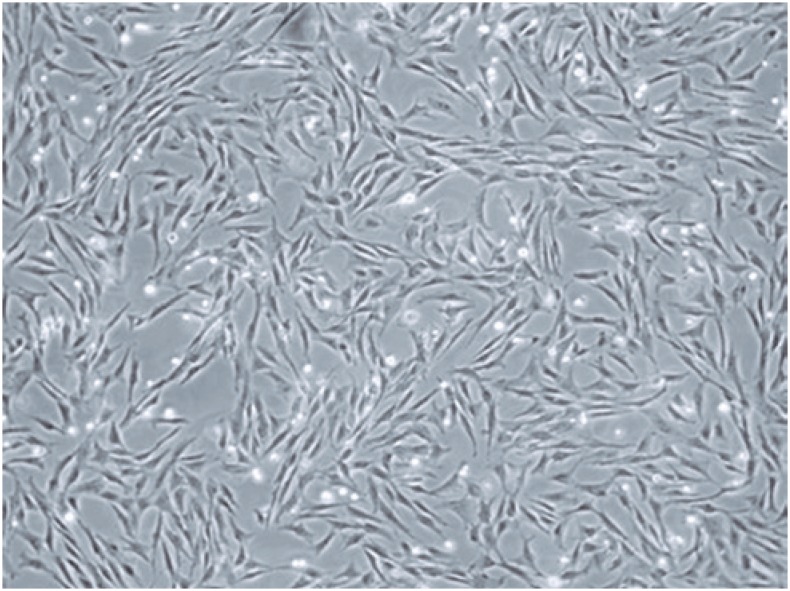
Fibroblast morphology of the multipotent adult progenitor cells

Every year, humanity has been increasingly affected by several conditions caused by biophysical or biochemical modifications, promoting imbalance in physiological systems (organs and tissues), and enabling, in the medium- and long-term, if not treated correctly, to lead to their failure. Recently, a new therapeutic approach has been presented in an extremely promising manner, called “stem cell therapy” or, more precisely, “therapy with multipotent adult progenitor cells” (MAPCs) (Figure 1). Known in the scientific field as mesenchymal stem cells (MSCs), the MAPCs have been the object of numerous investigations directed towards different types of diseases.

Multipotent adult progenitor cells is defined in scientific literature as an immature cell population, that is, unspecialized, capable of self-renewal and of originating multiple cellular lineages, according to the microenvironment in which it is situated. In 2006, the Mesenchymal and Tissue Stem Cells Committee of the International Society for Cell & Gene Therapy (ISCT) established minimal characteristics to identify human MAPCs: (a) cells adherent to plastic when maintained in standard culture conditions; (b) expression of CD105, CD73, and CD90, and lack of expression of CD45, CD34, CD14, or CD11b, CD79a, or CD19 and HLA-DR; and (c) capacity to differentiate into osteoblasts, adipocytes, and chondroblasts *in vitro.*

Present in all tissues of the body, MAPCs are responsible for maintenance of homeostasis and of tissue repair throughout life.^(^ ^1^ ^–^ ^3^ ^)^ Due to the capacity to migrate to damaged tissues by means of the chemotaxis process, MAPCs can be inserted and reach the site of the injury, by means of an intravenous infusion, promoting recovery of the damaged tissue. Some studies have shown that the majority of MAPCs transplanted into the body are not integrated with the damaged tissue, but exert their therapeutic effect by release of several trophic factors. These, on the other hand, support the repair process of the cellular microenvironment of the damaged tissues by cell differentiation process of endogenous MAPCs; the modulation of the immune system by means of the suppressive action of the innate and adaptive immune responses; cell renovation; inhibition of the oxidative stress responsible for leading to a reduction in the cell proliferation rate; increased senescence and inhibition of the MAPCS’ immunomodulating effect; the antiapoptotic action of preventing cell death, by restoring the local microenvironment; the production of antiapoptotic proteins, and antifibrotic action responsible for reducing the formation of scars, besides stimulating angiogenesis, a process by which new blood vessels appear from preexisting vessels.^(^ ^4^ ^,^ ^5^ ^)^

Nanotechnology is a multidisciplinary field that has developed rapidly over the last years. It was idealized by the physicist Richard Feynman, according to whom the atoms could be organized as necessary, as long as the laws of nature were not violated. Based on this scenario, the capacity to manipulate atoms and molecules, materials with new properties, could be synthesized, originating innovative products and processes. Such development tends to result in the manufacture of devices on a nanometric scale, ranging between 0.1 and 100 nanometers, applicable according to their physical-chemical properties to numerous areas of knowledge.^(^ ^6^ ^,^ ^7^ ^)^ Thus, Nanotechnology can be seen as the process of synthesis of functional structures on a nanometric scale, which results in devices with new physical, chemical, and biological properties, which depend on characteristics, such as structural size, conductivity, reactivity, functional conformation, and melting temperature.^(^ ^8^ ^)^

In the scope of Medicine, Nanotechnology arises as a new field of study, which proves to have great potential regarding treatment of various diseases. This new area of health is called “Nanomedicine” or “Bionanotechnology”, which can be applied in Medicine by using drug transport and release systems, robotic tools to be used in diagnostic and surgical processes, nanobiomaterials, gene therapies, nanobiomedical devices, among others.

Due to stem cells, more specifically MAPCs, and Nanotechnology being two extremely promising areas of science as to human health, a new field of study, called “stem cell Nanotechnology” gradually emerges. In this situation, Nanotechnology is used to assist stem cells to exert their therapeutic potential in the treatment, cure, and repair of injured tissues, in an effective and safe manner. Therefore, stem cell Nanotechnology has generated great interest, since it can result in a significant contribution to Regenerative Medicine and tissue engineering.

In the field of research of MAPCs, Nanotechnology has been used basically in three specific areas: (1) in marking procedures, which have the purpose of tracking the MAPCs after they are infused into the recipient, so as to generate data seeking a better understanding of the mechanisms involved in the processes of proliferation, differentiation, migration, and grafting in the host tissue;^(^ ^9^ ^)^ (2) in the transport of drugs, DNA, and microRNA introduced into the MAPCs for damaged organs and tissues; and (3) in the use of biomaterials that tend to reproduce the niche of the MAPCs, so they perform their therapeutic functions satisfactorily.^(^ ^10^ ^,^ ^11^ ^)^

## THERAPEUTIC PERSPECTIVES

**Figure 2:**
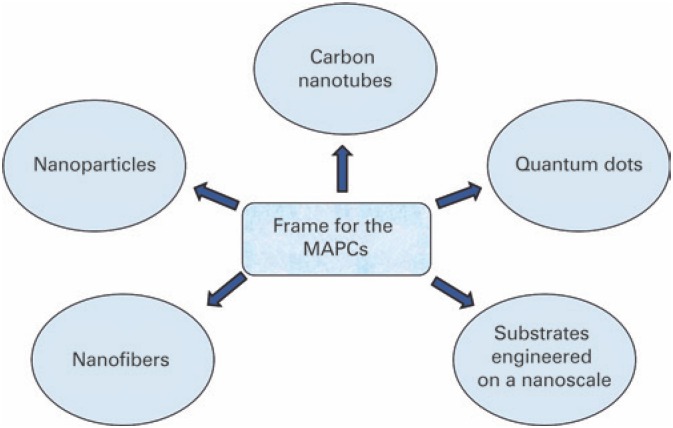
Materials and structures in nanoscale utilized as a scaffold so that the multipotent adult progenitor cells (MAPCs) can most effectively perform the process of cellular repair: nanoparticles, carbon nanotubes, quantum dots, nanofibers, and substrates engineered in nanoscale

Stem cells are considered the tool of the future in tissue engineering and Regenerative Medicine, and understanding the interaction mechanisms between nonmaterials and MAPCs is important for biomedical applications. Based on their nature, the most often used nanoparticles can be classified into five different groups:

– Carbon nanotubes: structures derived from graphene sheets, prepared by a precise control of orientation, alignment, length, diameter, purity, and density of the nanotube. They display adjustable chemical and mechanical properties, such as conductivity, biocompatibility, and nanoscale dimensions, serving as topographic lanes, besides generating electrophysiological properties. In the presence of a polycarbonate membrane and collagen sponges, they promote the osteogenic potential of the MAPCs.
– Inorganic base: ceramic-base nanoparticles, synthesized under a high temperature and pressure, formed by the combination of a metal and a non-metal component, with a structure that has high mechanical power and low biodegradability. Some examples are the nanoparticles of hydroxyapatite and tricalcium phosphate, which tend to promote bone formation.
– Metal base, in which the nanoparticles of metal oxide provide variability, exhibiting conductive or isolating characteristics. These display exclusive chemical and physical properties, with different charges in the center and at the extremities of the nanoparticle, having been used primarily in the tracking process of the post-transplant MAPCs. The MAPCs incubated with nanoparticles of magnetized ferrous oxide tend to promote the formation of calcium nodules in the presence of an osteogenic culture.
– Nanostructured hydrogels: substrates engineered on a nanoscale into three dimensions, carriers of drugs and proteins, capable of being introduced directly into the lesion site.
– Quantum dots: alternative to the use of organic stains and fluorescent proteins in the process of cellular marking and tracking *in vitro* and *in vivo,* as they are nanoparticles resistant to chemical and metabolic degradation, besides presenting with long-term photostability^(^ ^12^ ^)^ (Figure 2).

Among the greatest concerns related to the use of nanoparticles are their toxicity and environmental effects, since they can influence the processes of cellular adhesion, alignment, proliferation, differentiation, and migration.

### Osteoarticular diseases

One of greatest desires of Reparative Medicine is the reconstitution of bone tissue in cases of fractures, nonunion, and bone loss. One of the requirements for bone repair is the use of appropriate cell sources, carriers, and scaffolds, as well as specific growth factors to generate an environment biocompatible with the native bone structure.^(^ ^13^ ^)^

Although different approaches have been studied, aiming to obtain effective bone regeneration, distinct structural nanoscaffolding containing MAPCs have been used with great success.^(^ ^14^ ^,^ ^15^ ^)^ Researchers from the University of Tennessee analyzed the osteoinduction and osteoconduction effects of graphene-based materials (GBM) over the MAPCs *in vitro* and *in vivo.* The *in vitro* data demonstrated that the MAPCs maintained their morphology, their grade of adherence, and their viability in the presence of a scaffold made of GBM. After being implanted in bone lesions in rats, the MAPCs/GBM composition led to a marked improvement in bone formation and mineralization. These results demonstrated the applicability of the nanoparticles as adjuvant treatment for bone tissue engineering.^(^ ^16^ ^)^ Equivalent results were obtained by Jing et al., when using the carbon nanotube as scaffolding for the MAPCs, seeking more effective bone repair.^(^ ^17^ ^)^

Different from other tissues, the tendon and ligament have a low tendency to regenerate since they are composed of high-density extracellular matrix and reduced vascularization. When injured, the body tends to initiate a process of healing, which results in an inappropriate composition of the injured tendon and ligament. Due to its high porosity, the nanofiber allows a high inclusion rate of MAPCs in its structure, besides providing conformational alignment similar to what is found in the native tendon and ligament. Yin et al., reported the expression of specific tendon genes was significantly greater, similar to that of native tissue, when the MAPCs were inserted into the Poly (lactic acid) nanofibers; the fiber alignment was superior when compared to the control. The study demonstrated the generation of an adequate microenvironment tends to originate tendons and ligaments with desirable compositions.^(^ ^18^ ^)^

### Cardiovascular diseases

The idea of direct infusion of the MAPCs at the site of an infarction has resulted in functional improvements of the organ. Nevertheless, such an approach has proved limited due to the low survival rate of the transplanted cells in the damaged myocardium (less than 1% within one week after administration).^(^ ^19^ ^)^ The combination of Nanotechnology with the MAPCs has the purpose of increasing the cellular retention rate, from 1% to almost 100%, making myocardial regeneration more efficient. One strategy to increase the retention rate of the cells after the intramyocardial transplant, is to incorporate them into bioengineering nanomaterials capable of mimicking the three-dimension nanostructures present in the native tissue.^(^ ^20^ ^)^ There are two modalities of biological tissue engineering: adhesives for cardiac structure and injectable hydrogels. Both approaches represent viable options of treatment and the choice of the appropriate strategy of myocardial regeneration depends on the post-infarction time and size of the affected area. Cardiac adhesives, modified by tissue engineering, can be more useful for the complete substitution of noncontractile areas, while the injectable hydrogels containing MAPCs, or chemokines, or growth factors, aiming to attract endogenous MAPCs, have the advantage of representing a local and minimally invasive approach, besides minimizing the formation of scar tissue and attenuating the process of pathological remodeling.

Chachques et al., documented the myocardial area with infarction tends to present with structural modifications in the extracellular matrix of the myocardium, with decreased rates of type 1 collagens from 80% to 40%.^(^ ^21^ ^)^ Data obtained in a murine model demonstrated improvement in blood perfusion and reduced extension of the region with infarction, with increased ventricular wall thickness and angiogenesis, when the MAPCs - aggregated to a scaffold of type 1 collagens - were introduced.^(^ ^22^ ^)^

### Neurological diseases

Currently, the neurological disorders represent a challenging clinical problem for the medical community. Understanding the central nervous system functions and the development of new therapies aiming to repair damage caused to this system, after being affected by diseases and lesions have been the object of study of several groups of physicians and scientists. Since MAPCs are capable of originating several neural lineages *in vitro*, such as astrocytes, oligodendrocytes, and neurons, when transplanted *in vivo*, they tend to present a process of nervous tissue regeneration. The use of nanofibers and nanoscaffolds, along with the MAPCs, has shown more efficiency when compared to the results obtained by means of infusions with only MAPCs, since they demonstrate having important effects on the capacity of differentiating the MAPCs into nerve cells.^(^ ^23^ ^,^ ^24^ ^)^ Shah et al., demonstrated the capacity of a hybrid scaffold of graphene-nanofiber providing a favorable support to an effective differentiation of neural stem cells in mature oligodendrocytes without the need for inductive agents exogenous to the culture medium, such as viral vectors, growth factors and drugs. The study showed that a hybrid scaffold combining the morphological characteristics of the nanofibers and the properties of graphene, can be a powerful tool for the development of future therapies for diseases and lesions related to the central nervous system.^(^ ^25^ ^)^

### Tumors

Bearing in mind the known trophism of MAPCs for tumorigenic regions, affecting the biological characteristics of tumors, by means of release of growth factors, cytokines, and chemokines that tend to inhibit or promote growth of tumor cells, MAPCs have been considered as a potential cellular vehicle for directing nanoparticles loaded with anticancer drugs.^(^ ^26^ ^–^ ^28^ ^)^ This methodology seeks to increase the concentration of nanoparticles in the tumorigenic region in order to increase the efficacy of the drugs, reducing the side effects of medications.^(^ ^29^ ^)^ A study performed by Saulite et al., confirmed that MAPCs derived from cutaneous tissues are capable of carrying quantum dots nanocrystals - both *in vitro* and *in vivo* - to the tumor region, suggesting that this structure might be used as a transport mechanism for anticancer drugs.^(^ ^30^ ^)^ Saulite et al., demonstrated that quantum dots nanocrystals coated with carboxyl are biocompatible with the MAPCs obtained from the cutaneous tissue, which did not have the potential of proliferation, immunophenotype and differentiation affected due to the accumulation of quantum dots nanocrystals in the cells. In the presence of serum, the nanocrystals of quantum dots were internalized in the MAPCs by means of clathrin-mediated endocytosis, while in serum-free, the capture of quantum dots nanocrystals occurred by clathrin- and caveolin/lipid-mediated endocytosis. The study also showed the signal issued by the nanocrystals of quantum dots tends to decrease over time, likely due to the excretion of the quantum dots nanocrystals of the MAPCs. Such a fact tends to validate the potential of MAPCs as possible vectors that carry nanoparticles to tumorigenic regions.^(^ ^30^ ^)^

## CONCLUSION

The combination of Nanotechnology with the multipotent adult progenitor cells is a relevant and highly promising field, which may come to provide significant contributions for the treatment of several diseases. Nonetheless, the use of the multipotent adult progenitor cells along with the nanoengineered structures, with the objective of tissue repair, is still in its initial phases, and the performance of *in vitro* and *in vivo* research before this innovative technology becomes a reality for Medicine.
